# Experimental Evaluation of an Interferometric Light Microscopy Particle Counter for Titering and Characterization of Virus Preparations

**DOI:** 10.3390/v13050939

**Published:** 2021-05-19

**Authors:** Vesa Turkki, Elisa Alppila, Seppo Ylä-Herttuala, Hanna P. Lesch

**Affiliations:** 1 Kuopio Center for Gene and Cell Therapy, 70210 Kuopio, Finland; Elisa.Alppila@kct.fi (E.A.); hanna.lesch@kct.fi (H.P.L.); 2 Molecular Medicine Research Group, A.I. Virtanen Institute for Molecular Sciences, University of Eastern Finland, 70210 Kuopio, Finland; seppo.ylaherttuala@uef.fi; 3 Heart Center and Gene Therapy Unit, Kuopio University Hospital, 70210 Kuopio, Finland

**Keywords:** automated nanoparticle counter, titering, viruses, virus vectors, interferometric light microscopy, particle count

## Abstract

Virus particle concentration is a critical piece of information for virology, viral vaccines and gene therapy research. We tested a novel nanoparticle counting device, “Videodrop”, for its efficacy in titering and characterization of virus particles. The Videodrop nanoparticle counter is based on interferometric light microscopy (ILM). The method allows the detection of particles under the diffraction limit capabilities of conventional light microscopy. We analyzed lenti-, adeno-, and baculovirus samples in different concentrations and compared the readings against traditional titering and characterization methods. The tested Videodrop particle counter is especially useful when measuring high-concentration purified virus preparations. Certain non-purified sample types or small viruses may be impossible to characterize or may require the use of standard curve or background subtraction methods, which increases the duration of the analysis. Together, our testing shows that Videodrop is a reasonable option for virus particle counting in situations where a moderate number of samples need to be analyzed quickly.

## 1. Introduction

Virus particle concentration is critical for most research involving viruses, including virus-based gene therapy vectors, vaccines or virus-like particles. Several methods already exist to assess the total (full and empty) virus particle concentration, including enzyme-linked immunosorbent assay (ELISA) [1,2], high-performance liquid chromatography (HPLC) [3,4], transmission electron microscopy (TEM) [5,6], atomic force microscopy (AFM) [7], nanoparticle tracking analysis (NTA) [8,9,10], tunable resistive pulse sensing (TRPS) [11,12], super-resolution fluorescence microscopy (SRFM) [13], and techniques taking advantage of virus flow cytometry (“flow virometry”) [14,15,16].

**Figure 1:**
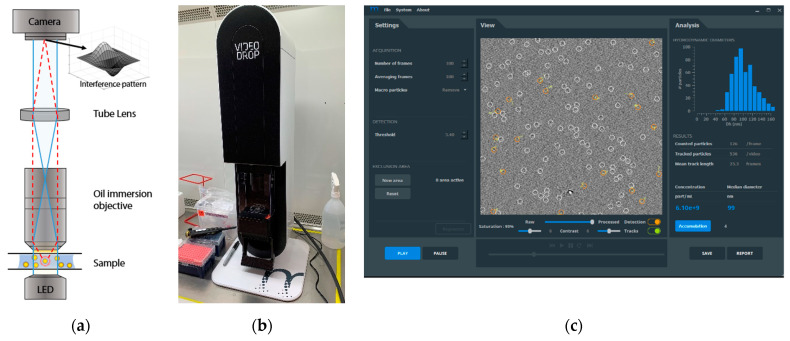
The videodrop system: (**a**) ILM (Videodrop, Myriade France) optical system; (**b**) the Videodrop device installed; (**c**) screenshot of the Videodrop software measurement view.

In 2016, Boccara et al. [17] developed a simple device based on interferometric light microscopy (ILM) for fast virus counting from environmental sources [18]. This technology was further developed and commercialized by the Myriade (https://www.myriadelab.com/, accessed on 19 May 2021) and is marketed as the Videodrop particle analyzer. Videodrop is able to measure >70 nm biological nanoparticles in solution from a single drop of a sample. Figure 1a shows the principle of the measurement, Figure 1b shows the device itself and Figure 1c shows the software measurement view. Videodrop is based on a transmission brightfield microscope used as a homodyne interferometer to detect, count and track nanoparticles (NPs). Counting particles from a defined volume directly leads to the concentration, while tracking the Brownian motion allows the measurement of their hydrodynamic diameter (D_h_) by using the Stokes–Einstein equation. The attractiveness of this method lies in its simplicity: the measurement is label-free, filtration-free, non-destructive and fast.

A Videodrop measurement consists of pipetting 5–10 µL of a sample on a glass coverslip, lifting the stage to the measurement position, operating the software and finally cleaning the device (several quaternary ammonium compounds approved). The sample is illuminated by a single light-emitting diode (LED) light source. A camera connected to the optical system records the particle movement. The user can adjust the particle detection threshold (TH) and various other settings—for example, specifying the tracked particle track length, adjusting the sample diluent viscosity, or selecting to discard macro particles from analysis. Adjustment may be necessary due to different intensity-signal levels (I_scatt_) scattered by the NPs depending on the material (e.g., refractive index, density) and the size of the particle. The device records short video blocs (100 frames, 140 frames per second) of particle movement and the user can record as many blocs as needed. Recording of a single bloc takes approximately 15 s and in the accumulation mode the device keeps recording until the user stops the measurement or the maximum number of blocs defined in the settings has been recorded. In our case, 5–15 blocs were generally recorded depending on the particle concentration. Increasing the number of blocs allows more particles to be analyzed and thus increases the precision of the measurement. As with any optical microscope, shot noise can degrade the image quality. Videodrop adjusts the saturation automatically, but the user needs to follow the saturation level indicator and start the measurements only once 90–95% saturation has been reached. Usually, this step does not considerably delay the start of measurements.

Each particle is classified either as detected or tracked, depending on whether it merely appeared for a moment (detected) or was followed for a longer period (tracked). Both detected and tracked particles contribute to the physical particles per milliliter (pp/mL) reading. The D_h_ is measured from the movement of tracked particles only.

The manufacturer recommends two alternative virus characterization techniques: the target concentration method or the visual threshold adjustment method. In the target concentration method, the threshold is pre-set beforehand during the assay development, the sample is serially diluted and the final result is calculated as an average from the results which fall on the linear part of the results curve (approx. 1 × 10^8^–7 × 10^9^ pp/mL, or 5–100 pp/frame, according to manufacturer). In the visual threshold adjustment method, no prior information regarding the sample is needed. The threshold is tuned visually until all particles are detected and the result can be read from this single measurement (range approx. 1 × 10^8^–5 × 10^10^ pp/mL, according to manufacturer). Generally, the target concentration method is suitable for defined assays, where high precision and reproducibility are needed, whereas the visual threshold method is more suitable for fast approximations of particle content, especially for comparing between samples without the need for absolute concentrations.

Here, we have tested the Videodrop particle analyzer for the titering of different viruses and virus-based gene therapy vectors. Both detection methods and three different viruses, lentiviruses (LV), adenoviruses (Ad) and baculoviruses (BV), were used in our tests. Our primary interest was to test the precision and reproducibility of the Videodrop system for a LV particle assay. Therefore, we first describe setting up an assay for lentivirus vectors, discuss its comparability with traditional titering methods, and then present brief examples on how the Videodrop performs with Ad and BV samples.

## 2. Materials and Methods

### 2.1. Virus Manufacturing, Titering and Characterization Methods

The used viruses were manufactured as described in previous publications [19,20,21,22,23,24,25,26]. The lentivirus vectors were of 3rd generation LV design [27].

The LV internal control (LV-GFP) was manufactured in 293T cells using the iCELLis^®^ 500 bioreactor as described by Leinonen et al. [20]. Shortly, cells were seeded into the fixed bed bioreactor and perfused using FBS-containing media (DMEM) until transfection. Transfection was performed 4 days later using the PEIpro^®^ transfection reagent from Polyplus. Before starting the virus collection, 24 h post-transfection, a complete media change using serum-free media was performed. Bioreactor runs were ended 72 h post-transfection. Bioreactors were drained and combined with the harvested media. The bulk harvest was endonuclease-treated with 30 U/mL benzonase (Merck) for 2 h at 37 °C in the presence of 2 mM MgCl_2_. The harvested material was treated as described by Valkama et al. [21]. Briefly, the harvested virus was clarified using the Millistak+^®^ Pod CE50, 0.77 m^2^ depth filter (Millipore) and a peristaltic pump. The LV product was further processed using the Mobius FlexReady tangential flow filtration (TFF) system (Merck) with the Sartocon^®^ Slice 100-kDa cassette (Sartorius). The virus was first concentrated 13-fold and then diafiltered into TFF buffer (50 mM HEPES + 300 mM NaCl, pH 7.5). No further processing was performed for the LV internal control and the material was aliquoted and frozen.

The Ad internal control is a replication-defective recombinant adenovirus vector. It is produced in iCELLis 500 bioreactor and clarified after harvest. Its downstream processing consists of crossflow ultrafiltration, two consecutive anion exchange (AEX) chromatography steps and a final tangential flow filtration. In addition to the internal Ad control, a wild-type adenovirus (Adenovirus Reference Material (ARM)) [28] was also used.

The BV samples were produced in Spodoptera frugiperda 9 (Sf9) suspension culture (110 rpm, 28 °C). Sf9 cells were grown for 3 days before infection with the stock virus. Infected cultures were grown for 4 days, after which the cell culture medium was clarified by centrifugation at 5000× *g* for 20 min at room temperature to remove cell debris. BV was purified and concentrated in two steps—first by overnight centrifugation (5000× *g*, 4 °C) through a 25% sucrose layer, and then by ultracentrifugation (25,000 rpm, +4 °C, 1 h) through a sucrose gradient (20–50%). The virus band was collected and dialyzed (Pierce 10 K MWCO cassette) by PBS at 4 °C.

**Figure 2:**
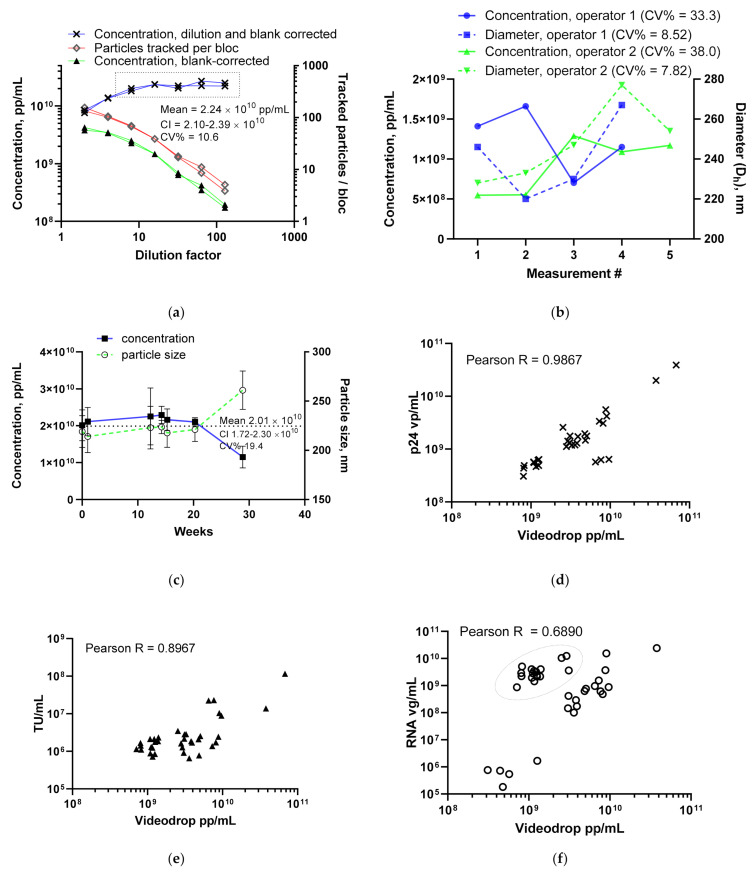
Videodrop LV assay results: (**a**) setting the LV assay range and minimum particle numbers. Two independent measurements of 2-fold LV dilution series (prepared fresh each time) were measured. The final blank- and dilution-corrected result as well as the blank-corrected raw reading and number of tracked particles are shown. The dotted line box shows dilutions falling within the set assay working range with their mean concentration, with the CI and CV% below. (**b**) LV repeatability results from a single 8× dilution as analyzed by two operators during a single day. (**c**) Intermediate precision results, measured from the same sample (different aliquot tubes) over several months. The mean is displayed as a dotted line, and CI and CV% are shown under the line. (**d**) Correlation between the Videodrop measurement and vp/mL p24 ELISA result. (**e**) Correlation between the Videodrop measurement and TU/mL qPCR result. (**f**) Correlation between the Videodrop and vg/mL ddPCR result. A group containing certain types of bioreactor samples showing a lower pp/mL value than expected based on the RNA genome titer is circled using a dotted line.

For the various (*n* = 41) LV samples used in Figure 2d–f, both adherent and suspension bioreactors as well as flask production techniques were used for the upstream process. Of these 41 LV samples, 29 were non-treated harvest samples and 12 were downstream processed or at least clarified by depth filtration or centrifugation and/or benzonase treated. The downstream processing consisted of tangential flow filtration and/or chromatography steps or ultracentrifugation. The empty LV virus-like particle (VLP) preparation was produced in cell culture flasks and concentrated using ultracentrifugation.

The viruses were titered by applying established methods [20,29]. Briefly, the LV particle titers were analyzed using a p24 ELISA kit (Abcam, cat# ab218268) according to manufacturer’s instructions. LV genome titers were analyzed using a RT-ddPCR assay with WPRE primer/probe set. LV transduction unit (TU, units leading to marker gene expression) titers were analyzed by transducing HeLa cells with the sample, extracting all DNA after 3 days and quantitating the number of cell genomes and LV transgenes (CMV primer/probe set) using a qPCR assay. Ad and BV virus genome (vg) titers were analyzed using a ddPCR after DNase and Proteinase K treatments (CMV, hexon and WPRE primer/probe sets). Ad HPLC titer was analyzed by our collaborator using a validated HPLC assay based on Shabram et al. [3]. The titers for the control viruses used in the study for LV, Ad and BV dilution series are shown in Table 1. Ad TEM diameter was measured from negatively stained samples using the MiniTEM (Vironova AB) system and an automated script for Ad recognition. 

### 2.2. Videodrop Measurements

**Figure 3:**
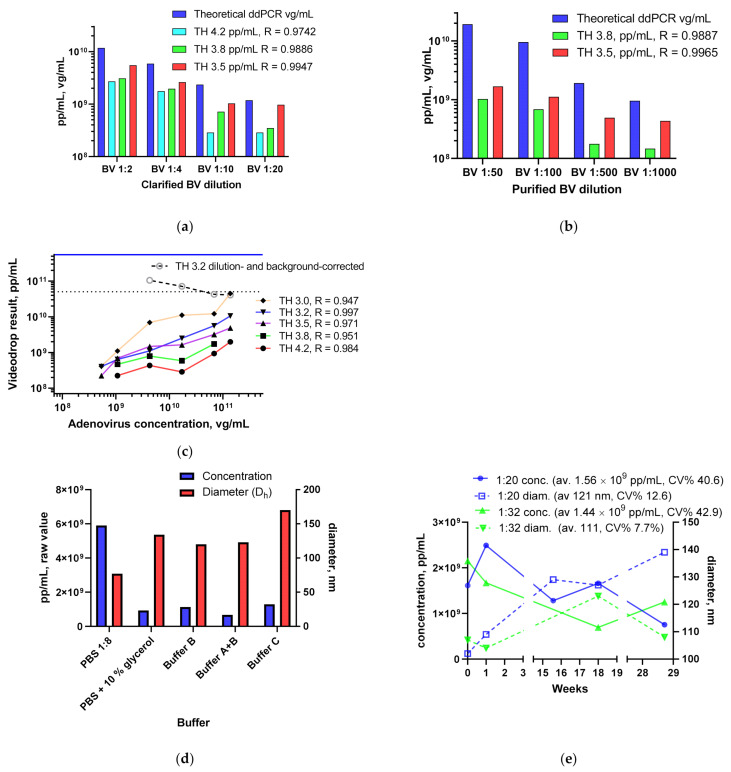
Blank-corrected Videodrop BV and Ad measurement results: (**a**) clarified BV sample dilutions (PBS) were measured using the standard setting (threshold 4.2) and reduced threshold values (3.8 and 3.4). Blank-subtracted values were compared to the ddPCR vg/mL titer (titered from the undiluted sample). (**b**) Purified BV sample dilutions (PBS) were measured using threshold values 3.8 and 3.5. (**c**) Adenovirus dilution series was measured using different detection threshold values. Raw values and the dilution-corrected value for TH 3.2 are shown. Dotted line shows the upper raw value concentration limit set for the manufacturer for the visual threshold adjustment method. The solid blue line shows the virus genome titer. Correlation coefficients between the vg/mL and pp/mL concentrations shown in the legend. (**d**) Adenovirus samples (6.85 × 10^10^ vp/mL) diluted in different buffers. Buffer A and B are Ad chromatography buffers, and buffer C is a formulation buffer. (**e**) Repeatability results (raw values, TH 3.5) for an Ad sample (separate aliquots) measured over several months.

All the measurements described in this work were recorded with the accumulation mode and with the removal of macro particles enabled (removal settings: min. radius 10 and min. hot pixels 80). For the LV serial dilution analysis and for testing against the traditional titering methods, the target concentration method was used: 3–5× PBS (Gibco, 14190-094) dilution series was performed, and 7–8 µL sample was measured on the Videodrop device (software qvir: 2.5.3.6443/Mil-1, updated to 2.5.5.6797 for the work summarized in Figure 3b and Figure 5e). Readings falling inside the assay range were blank-subtracted and used to calculate the final result. As an exception from the target concentration method, the LV single dilution repeatability results were measured using a single 8× dilution only. The default threshold (TH) value of 4.2 was mostly used for the lentivirus measurements shown in this paper. As an exception, a part of the intensity values shown in Figure 5d were recorded using TH 3.8. A total of 5–20 blocs were recorded per sample. 

LV degradation tests were performed by adding Triton X-100 (Sigma, ×100) to a final concentration of 0.1% [31,32,33]. The first sample was immediately measured after Triton X-100 addition and the rest after 10, 15 or 25 min incubation.

Ad and BV analysis were performed using varying TH values, as shown in the figures. Some Ad results are shown as raw values (indicated the figure caption). Ad heat-treatment was performed on a +47 °C heat block, on which separate tubes were placed for different incubation durations.

Ad ammonium sulfate aggregation was performed by mixing the Ad stock 1:8 with PBS containing 40% (NH₄)₂SO₄ (Sigma, A4418). Samples were measured (TH 3.2, 10 blocs) after 5, 20 and 40 min.

For the buffer effect test, Ads were diluted 1:8 in the studied buffers. Buffers A and B are typical high- and low-salt HEPES-based buffers used during Ad downstream processing for a 300–600 mM NaCl gradient, according to Burova and Ioffe [34]. Buffer C is a sodium phosphate-based formulation buffer containing 10% glycerol. For the cell lysate intensity and diameter tests, the adenovirus stock was first mixed at a ratio of 1:5 with cell media, which was then mixed at a ratio of 1:2 with a cell lysate containing 1.1 × 10^10^ lysed cells/mL. The final sample therefore contained 5.48 × 10^10^ vg/mL of lysate from 5.5 × 10^9^ cells/mL.

Normal device cleaning between the measurements consisted of sample removal using a lint-free wipe. The sample-contact parts (objective glass and the lens protective window) were then cleaned using 70% ethanol.

### 2.3. Calculations and Statistics

After measurements, the majority of the results were saved both as a PDF report and as a CSV file, of which the former was a back-up copy and the latter was used for the analysis. Results were analyzed using GraphPad Prism 8.4.2. Unpaired *t*-tests (parametric test) were used to determine potentially significant differences between two datasets. Pearson’s correlation coefficient was used to measure the correlation between Videodrop results and traditional methods. Confidence levels and confidence intervals were set to 95%. The ROUT method [30] was used to assess outliers in the ddPCR vg/mL data (Q = 1%). 

## 3. Results

### 3.1. Lentivirus Particles/mL Assay Development

**Figure A1:**
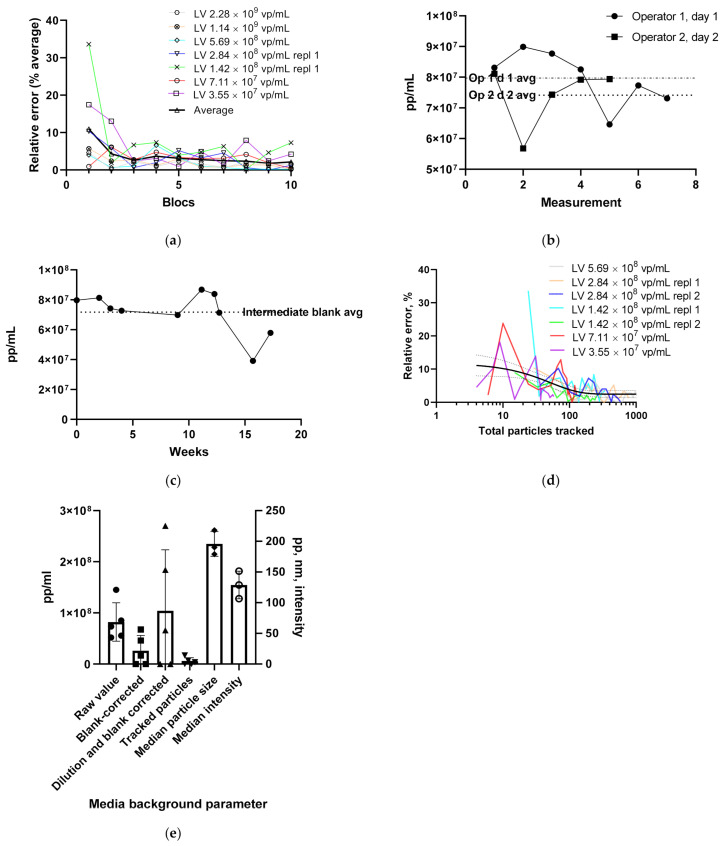
Requirements for measurements were studied by measuring a 2-fold lentivirus dilution series: (**a**) the effect of the measurement duration (number of blocs) on the relative error of the particular bloc. (**b**) Blank (PBS) repeatability was tested by two operators on different days by performing several blank measurements. Normal device cleaning was performed after the measurements. Operators’ averages are shown in the figure with dotted lines. (**c**) Intermediate blank average was followed by recording either the daily blank result or the average in case of multiple measurements. Intermediate average is shown in the figure as dotted line. (**d**) The minimum number of tracked particles was studied by measuring different LV dilutions and calculating the relative error after each bloc against the final result. An exponential one phase decay equation for with 95% CI is shown as a black line. (**e**) The level particles of cell culture media background were studied in HEK-derived cells 2-3 days after seeding on two separate days.

The preliminary work for LV particle assay set-up is described in Appendix A. First, it was necessary to define the minimum length (blocs) of a measurement (Figure A1a) and the minimum number of particles tracked (Figure A1b). In order to assess the background particle level, PBS blank intra-day repeatability (Figure A1c) and its intermediate average (Figure A1d) were determined. Based on these tests, at least 5 blocs and 40 tracked particles should be measured for a LV dilution to be included in the pp/mL results. In order to ensure that the size distribution histogram is representative of sample size distribution, the operator should record at least 100 tracked particles. A detection limit (limit of detection, LOD) was set to 1.1 × 10^8^ pp/mL (before blank subtraction) based on the PBS blank intermediate average and standard deviation ((SD) average + 3SD). It was also noted that the background signal from the used cell culture media (in 1:4 dilution, Figure A1e) can affect the lowest results. Cell culture media led to particle counts with a raw value and blank-corrected mean of 8.22 × 10^8^ (CV% 45.7) and 2.67 × 10^7^ (CV% 115) pp/mL, respectively. It should be noted that some media measurement replicates fell below the PBS blank average, thus resulting in a zero pp/mL result.

#### 3.1.1. LV Dilution Curve and LV Control Intermediate Precision

With the preliminary assay limits in place, a 2-fold LV dilution series made of internal LV-control (8.29 × 10^9^ vg/mL) was analyzed for its particle titers. The aim was to set the working range for the LV assay. Two independent dilution series preparations and measurement rounds were performed on different weeks (Figure 2a). We observed a loss of linearity at low dilution factors (2 and 4×). Blank-corrected particle concentrations above approx. 3 × 10^9^ pp/mL and over 100 particles tracked per bloc led to lower dilution-corrected particle counts. The two lowest dilutions, 2 and 4×, had 48.5% recovery, calculated from the average value of higher dilutions (unpaired *t*-test, *p* = 0.0035). Based on this test, dilutions leading to raw LV concentrations between 1.1 × 10^8^ and 3 × 10^9^ were confirmed suitable for the target concentration method. 

The repeatability of an LV concentration measurement was assessed from a full LV assay as well as from a single dilution. For the single dilution repeatability, the 8× dilution preparations and measurements were repeated by two operators on a single day (Figure 2b). Other samples were also measured between the repeatability test samples. The repeatability (CV%) for a single dilution was 35.7%. It should be noted that two types of non-routine operations, sample stage height adjustments and cover glass changes, were performed between the test samples. Full assay repeatability was performed by a single operator by repeating the assay three times on a single day (3.50 × 10^10^, 2.50 × 10^10^ and 2.87 × 10^10^ pp/mL, $\bar{x}$ = 2.96 × 10^10^ pp/mL, CV% = 14.0%).

Intermediate precision results were gathered over several months by repeated assays of the LV internal control by two operators (Figure 2c). The LV internal control was used for two reasons. First, it has been extensively titered for its p24 particle titer and virus genome copies (Table 1), and second, as a downstream intermediate, it represents a typical LV sample for which the Videdrop is used. The mean physical particle titer, 2.01 × 10^10^ pp/mL (CI 1.72–2.30 × 10^10^, CV% 19.4), was higher than either the virus particle (4.55 × 10^9^, CI 4.16–4.93 × 10^9^) or virus genome (8.29 × 10^9^, CI 6.85–9.74 × 10^9^) titer. The internal LV control is a depth filter-clarified and TFF-processed virus. It has not been chromatographically purified and thus still contains cell-derived impurities. However, due to the nature of the LV and the relatively high number of non-virus material in LV preparations, a matching result with existing titers was not expected. Additionally, a drop in the mean internal control titer from 2.1 × 10^10^ vp/mL to 4.55 × 10^9^ vp/mL was seen when the earlier p24 ELISA kit by another manufacturer was changed to the current one. The 29 weeks timepoint in Figure 2c appears to deviate markedly from other observations. The drop in the titer is associated with an increase in the particle size, and therefore sample aggregation cannot be ruled out.

#### 3.1.2. Comparing the LV Assay against Traditional Titers

With the main LV assay specifications (LOD, working range, dilution scheme) in place, various LV samples with different transgenes and pseudotypes were analyzed using the Videodrop system. The samples included upstream and downstream materials harvested from cell culture flasks as well as from adherent and suspension bioreactor runs. The full titer set (virus particles, virus genomes, transduction units) was available for 27 out of 41 samples. Videodrop results were plotted against the in-house titering results from p24 ELISA vp/mL particle titer (Figure 2d), functional qPCR TU/mL titer (Figure 2e) and virus genome RT-ddPCR vg/mL titer (Figure 2f). Videodrop measurements showed a strong correlation with all three of the in-house titering methods. A specific type of bioreactor upstream sample (circled in Figure 2f) displayed lower than expected titers in Videodrop compared to the in-house vg/mL assay, but the same issue is seen in relation to vp/mL p24 result, and is therefore not Videodrop-specific (data now shown). As expected, the p24 vp/mL particle titer showed the best correlation with the Videodrop result.

Timewise, the Videodrop device offers superior time to results compared to traditional ELISA or PCR assays when a low or moderate number of samples (approx. 1–40 tubes) need to be measured. A single visual threshold measurement takes only a couple of minutes. On the other hand, analyzing a large number of samples becomes a slow process with a long hands-on time. As an example: a target concentration method session, during which 40 tubes (8 LV samples, 5 dilutions each) were carefully analyzed, took a total of 4 h. The time consumption was the following: 30 min for planning, set-up and sample retrieval; 30 min for tube marking, documentation and dilution series; 3 h for sample measurements and handling the data. Each dilution was measured in less than 3 min, with an additional 1.5 min for data saving and cleaning between the tubes.

#### 3.1.3. BV and Ad Tests

In addition to LV assay development, Videodrop was also tested using BV and Ad samples. A clarified BV stock was analyzed using a ddPCR virus genome assay for its virus genome content and the sample was later measured with Videodrop (Figure 3a). It was discovered that the blank-corrected Videodrop results underestimated the virus titer with the standard threshold setting (TH4.2), but it could be corrected to some extent by threshold adjustments. Improved correlation was achieved by reducing the detection threshold value. A strong correlation over the tested range was seen using the lowest tested threshold value of 3.5. Similar results were seen with a purified and concentrated BV sample (Figure 3). With this sample, the titer underestimation was even more pronounced.

Due to its smaller size, and resulting lower intensity values, setting a threshold for adenovirus detection required a compromise (Figure 3c). Based on visual estimation of the true interference signals, the threshold should be set at between 3.4 and 3.8, yet the best correlation was achieved using TH 3.2. TH values between 3.2 and 3.5 were used for the results below. Despite the strong correlation coefficient and good linearity observed when using TH 3.2, the dilution and background-corrected Ad pp/mL titer was lower than the virus genome titer or virus particle titer. When all dilutions exceeding the LOD from Figure 3c were averaged, the internal Ad control titer was 6.49 × 10^10^ pp/mL (CI 3.93–9.06 × 10^10^, CV% 40.3). The lowest dilutions with well-correlating TH 3.2 would yield results very close (up to 4.12 × 10^11^ pp/mL) to the expected value (5.48 × 10^11^ vg/mL), but the raw particle concentration was under the LOD level and background subtraction led to zero pp/mL. In order to make sure that the difference is not due to the internal Ad control, data from another experiment (TH 3.4 with 100×, 20×, 10× and 5× dilutions) using the well-characterized ARM adenovirus reference material (ATCC^®^ VR-1516™) [28] was checked and the background- and dilution-corrected pp/mL titer was also lower than the HPLC virus particle titer (HPLC 5.8 × 10^11^ vp/mL, Videodrop 2.83 × 10^10^ pp/mL).

Despite the routine 4× or higher dilution with PBS, we also tested the effect of the buffer when non-PBS diluted sample was used. The diluent was expected to play a role at least for the particle diameter, as these buffers have different viscosities than PBS, and this has a direct effect on the D_h_ through the Stokes–Einstein equation. Ad samples were mixed with different buffers and measured (Figure 3d). With no technical repetitions, strong conclusions cannot be drawn, but it appears as if the used diluents all reduce particle concentration and increase the particle diameter. The effect on particle concentration was unexpected and could be caused by either loss of Ads due to aggregation, or due to buffer interference. A minimum of 4× PBS dilution was set in the LV concentration assay.

Ad intermediate precision was determined over several months by performing single-dilution measurements (Figure 3e). For TH 3.5 and 1:20 dilution, for which the most repeats (*n* = 5) are available, the mean, CI and CV% are 1.56 × 10^9^ pp/mL, 7.72 × 10^8^–2.35 × 10^9^ and 40.6, respectively. LOD for the Ad and BV samples (before background subtraction) with TH3.5 is 1.3 × 10^9^. The particle median diameter from Videodrop for an Ad sample (121 nm in the aforementioned TH 3.5, 1:20 data) agrees with the dynamic light scattering diameter values for the same virus (approx. 115–125 nm, data not shown).

#### 3.1.4. Using the Videodrop System for Detecting Virus Breakage or Aggregation

**Figure 4:**
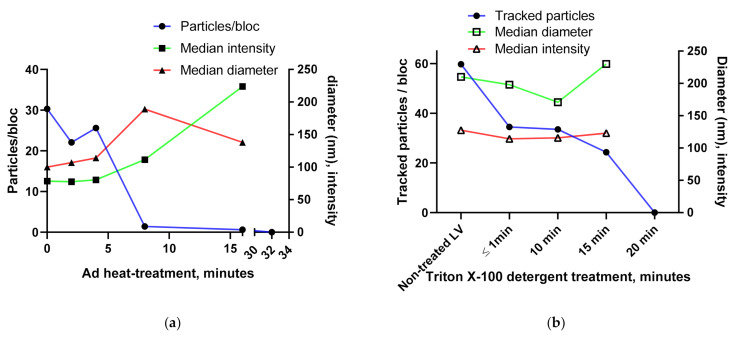

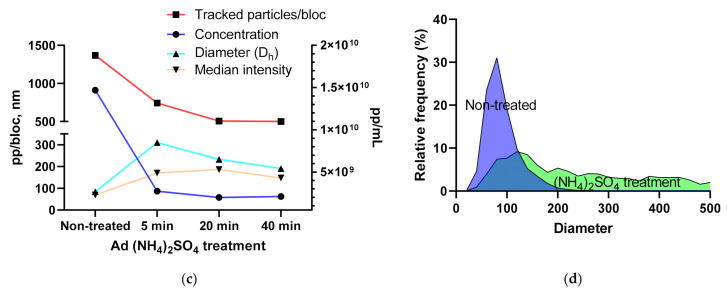
Virus breakage and aggregation tests using Ad and LV: (**a**) Ad heat treatment using a temperature sensitive (pIX deleted) Ad. (**b**) Lentivirus chemical inactivation using Triton X-100 treatement. (**c**) Ad aggregation test using ammonium sulfate (**d**) The change in particle diameter during the virus aggregation test. The 5, 20 and 40 min treatments were combined into a single data set for the visualization.

We wanted to test if Videodrop could be used to detect virus breakage and aggregation. A heat-sensitive (pIX-deleted) adenovirus preparation was subjected to heat-treatment at +47 °C (Figure 4a). Based on earlier experiments and supporting literature data from +48 °C [35], we know that the particular virus almost fully loses its infectivity as measured by our infectivity assay after 20–40 min at +47 °C (data not shown). Samples were analyzed (TH 3.5) after 2, 4, 8, 16 or 32 min heat treatments. Very few particles were tracked at 8 (95.4% decrease) or 16 min (98.0% decrease), with the particle count reaching zero at 32 min. An increase in particle intensity and D_h_ was also seen, possibly due to aggregation of the broken virus debris. Here, the drop in the tracked particle value appeared as a useful measure of the virus breakage, as the observed particle value seems prone to background signals from broken viruses.

A similar virus inactivation experiment was performed using LV (Figure 4b). The well-characterized Triton X-100 inactivation [31,32,33] was performed and results were read immediately and after 10, 15 and 20 min of treatment. The tracked particle number dropped to zero in 20 min, as expected based on the literature data.

The ability to analyze the extent of virus aggregation is an important factor in virus vector research and development. To test how Videodrop results react to Ad precipitation and aggregation, we used ammonium sulfate [36] to precipitate the virus (Figure 4c). Addition of the ammonium sulfate led to a major reduction in particle concentration and to a reduction in tracked particles. Additionally, an increase in the particle diameter was seen and, to a lesser extent, an increase in particle intensity. When the particle sizes were visualized (Figure 4d, non-treated vs. all treatments combined), the loss of the single particle Ad peak (virus diameter approx. 73–83 nm [37]) and the appearance of higher diameter structures was evident.

#### 3.1.5. Intensity and Diameter Data 

**Figure 5:**
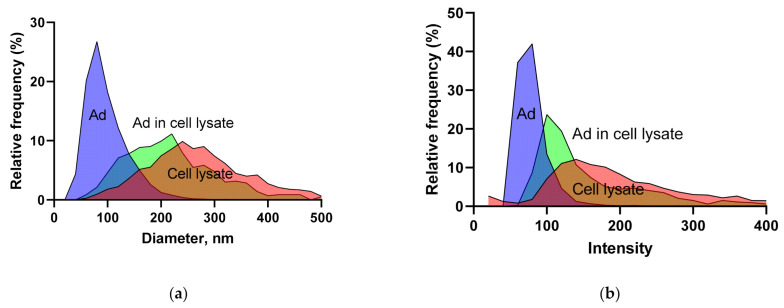

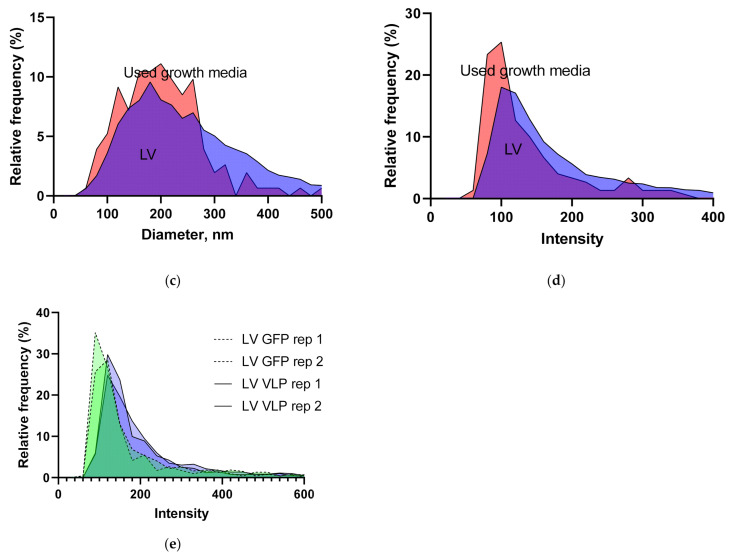
Intensity and diameter data from Ad and LV preparations (data pooled from several measurements), together with a common source of background signal. (**a**) Diameter of Ad, cell lysate and the two combined. (**b**) Intensity of Ad, cell lysate and the two combined. (**c**) Diameter of LV and used cell culture media. (**d**) Intensity of LV and used cell culture media. (**e**) Two different measurements of LV internal control (dotted line) and a LV VLP (empty LV, solid line).

As Ads are commonly harvested by lysing the cells, we used the Videodrop to study the diameter (Figure 5a) and intensity (Figure 5b) values of adenoviruses, cell lysate and a mixture of the two. The Ad peak diameter agrees well with the literature value [37] and with that determined for the same virus vector from three different batches using an electron microscope with automated particle recognition [15] (75.5 nm, *n* = 10,742, CI = 75.4–75.6, CV% = 7.71).

Additionally, experimentally purified lentiviruses (data combined from two differently processed samples) and growth media after 2–3 days on cells were studied for the diameter (Figure 5c) and intensity (Figure 5d) values. The peak overlap was notable, as expected, as the cells release a lot of particulate, including extracellular vesicles, into the growth media. As the particle concentration in the used cell culture media is, however, considerably lower than the concentration of a good lentivirus harvest material, the overlap is not a major issue. We also compared the LV internal control against an empty LV VLP preparation (two measurements each) to see if empty LV particles could be differentiated from the full particles based on their intensity. Considerable peak overlap was observed, and the minor differences could be due to the different production processes (clarification and TFF vs. ultracentrifugation).

## 4. Discussion

In this manuscript, the Videodrop nanoparticle analyzer was evaluated. To our knowledge, this is the first manuscript describing Videodrop’s performance in virus vector sample titering and characterization.

Despite the Videodrop method being non-specific for viruses, the obtained results are generally in line or correlating with the traditional virus-specific titering methods. More specifically, we have shown herein that the Videodrop can be successfully used for the analysis of LV samples provided that the background signal level is carefully controlled. Due to the large concentration of extracellular vesicles released by mammalian cells, the background signal correction is especially important for low concentration upstream samples. 

The intermediate precision (the precision over extended time in real-life conditions, different diluent batches, different operators, etc.) for the LV particle concentration assay was 19.5% (CV%). The repeatability (intra-day precision) for the LV particle concentration assay was 14.0%. Repeatability was also tested by repeated sample preparations and measurements as a single dilution in a “worst case scenario”, with device adjustments, a cover glass change and other samples being measured between the test samples. The single dilution repeatability (35.7%) and intermediate precision (19.4%) values are therefore not comparable in this case, as the repeatability should usually give the smallest variation.

A wide variety of LV sample types were successfully tested. Due to the nature of the LV, virus preparations always also contain non-viral particles and therefore fully corresponding results were not expected, nor did we see the need to finetune the TH value for LV at this stage. The correlation between Videodrop and the traditional p24 ELISA assay results was very good. Furthermore, this manuscript demonstrates Videodrop’s ability to measure BV and Ad concentrations, yet the pp/mL titers are underestimated and the threshold value needs careful adjustment. Table 2 summarizes the key figures of LV and Ad assays. Physical particle titers for Ads (internal Ad control, and the ARM [28]) were lower than their genome (ddPCR) or virus particle (HPLC) titers, but the correlation between the titers was strong, which is usually enough for effective utilization of a titering method.

Videodrop can be used to measure >70 nm physical particles from biological samples. The minimum virus particle observable with Videodrop is thus slightly above that of the NTA, TRPS or flow virometry. TEM or super-resolution imaging-based systems reach considerably better resolutions but are not suitable for fast virus quantification. 

In principle, the virus size is a major determinant of the optimal TH value. Smaller viruses should generally require lower threshold settings, yet we also saw a need to reduce the threshold from its default setting when analyzing larger BV, with enveloped, rod-shaped capsids with a length of 230–385 nm and a diameter of 40–60 nm. The threshold adjustment raises an obvious concern. Lowering the threshold increases the number of detected and tracked particles (background), even from pure buffer samples. In theory, this will lead to lower threshold measurements appearing less pure due to the increased background signal in the intensity and diameter histograms, and therefore a background subtraction option for automatic concentration, intensity and density correction would be a useful feature. 

Virus diameter and intensity values can be used to further characterize the sample. The variation coefficients were lower for the diameter values than for the particle concentration, making the median diameter results quite stable. After measuring Ad mixed with cell lysate from a HEK293 cell pellet in PBS, it became evident that excluding the cell lysate signal based on the diameter or intensity value would not be a trivial task due to the overlap of the peaks and the variability of the results. The lysate, however, contained an excess of cells compared to a typical Ad lysate, and therefore the signal separation could be easier with the real samples. The shape and location of the peaks can be used to estimate the purity of the virus preparation. The sample buffer composition affects the diameter (and perhaps also the concentration). Therefore, a minimum dilution into a standard buffer may be a practical solution.

Any microscopic object can screen nanoparticles behind it. Particles with significant size differences can create signals with too high differences in amplitude for the camera dynamics to resolve. Together, these two phenomena are the main factors behind the “screen effect”, which becomes significant when the concentration of microscopic objects and nanoparticles is high enough. The data obtained here are not sufficient to determine the level of screen effect for Videodrop measurements. In theory, ILM is less affected by the screen effect than various other techniques relying on NPs’ scattered light (e.g., NTA). In an interferometric observation method, the detected signal is proportional to √I_scatt_, not I_scatt_, as in non-interferometric techniques [38]. Therefore, when two types of particles have a 10^6^ ratio (fold difference) in their scattered light, the signal ratio in ILM is only √10^6^ = 10^3^. The camera used in ILM also differs from that used in dark field microscopy (e.g., NTA), giving it a high dynamic range and reduced saturation likelihood. However, our studies show that too concentrated samples lead to underestimated virus titers (due to screen effect or not), highlighting the importance of setting the working concentration limits. Low-concentration samples with impurities are also problematic to analyze, due to the high background signal level and because the majority of the measured particles may be non-viral (e.g., cell debris, media components, extracellular vesicles). Analyzing only a specific population of particles, and thus excluding most of the non-viral material, based on the intensity or diameter values may be possible in some cases, but we did not see that as a viable option for our LV preparations. Because of the background noise from cell debris, the device has not been used to analyze Ad from crude cell lysates. According to our estimates, the cell debris-derived particles would require diluting the sample more and lead to highly variable results. The techniques utilizing virus-specific staining, such as flow virometry [15] or NTA [10], can better overcome the impurity problem.

The dual mode of detection (tracked and counted particles) leads to some obvious problems, especially regarding the very high background signal level in a situation where a low threshold (e.g., TH 3.2) is being used. Pure PBS may give a fairly high titer (e.g., 4.5 × 10^8^ pp/mL) based on detected particles, despite the fact that the number of tracked (and thus more likely real) particles may remain zero, whereas a low-concentration virus preparation may have practically the same pp/mL titer with tracked particles found. This begs the question as to whether the tracked particle vs. detected particle ratio should remain within a specified range in order to report the result as “approved”. The high background noise is a significant limitation for the device usage as certain sample types fall under the set LOD. According to our communication with the manufacturer, these issues have been recognized and are being remedied.

Regarding the practical usage of the device, we feel that it is relatively easy to use following brief training. Use is easiest in a situation where pre-set values for the threshold and detection limits exist a priori. The time to results for a single sample is rivalled only by flow virometry, as results can be obtained in a matter of minutes. By comparison, the NTA is also reasonably fast, whereas microscopy-based techniques such as TEM or SRFM require tedious sample preparation and time-consuming data analysis. Traditional assays may still offer faster options, e.g., when analyzing tens or hundreds of samples. The time to result depends very much on whether the test sample is a dilution series or a single tube. In our case, measuring 40 LV tubes (8 LV samples with five dilutions each) using Videodrop was still faster than a p24 assay, but anything more may have taken longer overall. When comparing the costs of the different systems, the price of the device, its maintenance and consumable costs should be considered. The Videodrop device has a reasonable price and price-per-sample is very low, as there are no stains or consumables that would need to be exchanged after each measurement and the diluent price is generally negligible.

Various analytical techniques exist to quantify the total physical particle or virus concentration. See Table 3 for comparison of some of the commercially available NP counting devices for rapid total virus particle quantification. The field is rapidly developing and it remains to be seen which technologies become the main analytical tools among the scientists and gene therapy vector manufacturers.

## 5. Conclusions

We have evaluated the Videodrop nanoparticle counter by means of developing an LV particle concentration assay. Furthermore, we have tested the system using Ad and BV. Following these tests, we conclude that ILM and the Videodrop device are suitable tools for analyzing the particle concentrations of several different types of lentivirus preparations, of clarified or purified BV preparations, and purified adenovirus preparations. 

## Figures and Tables

**Table 1:** The control viruses used in the study.

Vector/Virus	Titer Type	Titer Value	CI	*n*	Method
LV internal control	vp/mL	4.55 × 10^9^ *	4.16–4.93 × 10^9^	46	p24 ELISA
	vg/mL	8.29 × 10^9^	6.85–9.74 × 10^9^	49 **	WPRE ddPCR
	TU/mL	8.76 × 10^6^	7.59–9.93 × 10^6^	146	qPCR
Ad internal control	vp/mL	5.0 × 10^11^	NA (external data)	NA	HPLC
	vg/mL	5.48 × 10^11^	4.33–9.81 × 10^11^	22	CMV ddPCR
Purified BV	vg/mL	9.58 × 10^11^	7.60 × 10^11^–1.16 × 10^12^	1	WPRE ddPCR
Clarified BV	vg/mL	2.35 × 10^10^	1.69–3.02 × 10^10^	1	WPRE ddPCR

* A previous p24 ELISA kit from another manufacturer gave this vector a long-term average titer of 1.7 × 10^10^ vp/mL (CI 1.3–2.1 × 10^10^, *n* = 9), which is more realistic in light of the genome titer. ** Two outliers (calculated using the ROUT method [30], Q = 1%) were removed from the data, most likely arising from a laboratory error or a device malfunction. N is the number of independent titerings, each consisting of multiple technical replicates. CIs were calculated from the averages of independent titerings if *n* > 1, and otherwise technical replicates were used.

**Table 2:** Characteristics of Videodrop performance for real-life LV and Ad measurements.

Vector/Virus	LV	Ad
Threshold setting	4.2	3.2–3.5
LOD, pp/mL (blank av + 3SD) *	1.1 × 10^8^	1.3 × 10^9^ (TH 3.5)
Working range upper limit, pp/mL	3 × 10^9^	N.D.^†^
Repeatability, single dilution (CV%) ^‡^	35.7	N.D.
Repeatability, full assay (CV%) ^§^	14.0	N.D.
Intermediate precision, full assay (CV%) ^¶^	19.4	40.6

N.D. = not determined; * Before blank subtraction; ^†^ 1.05 × 10^10^ is the highest Ad concentration tested using TH 3.2. We assume the working range upper limit to be considerably higher; ^‡^ repeated measurements of a single dilution by two operators on a single day. Device adjustments, a cover glass change and other samples were measured between the test samples; ^§^ repeated full assay by a single operator on a single day; ^¶^ repeated full assay by two operators over several months.

**Table 3:** Comparison of commercially available devices for total nanoparticle counting with rapid time to results.

Method, Devices	Description	Range	Advantages	Disadvantages	References
**Interferometric light microscopy (ILM)** Videodrop (Myriade)	A drop (5–10 µL) of sample is illuminated by a visible light LED. Transmission brightfield microscope is used to measure the interferometric signal for NP detection. Brownian motion tracking for D_h_.	>70 nm for biological NPs	Fast (≥3 min) and easy to use. Low sample volume Less affected by the screen effect No fluidics → easy start-up, cleaning and maintenance. Affordable device, low price-per-sample	High background noise Concentration limits max. 1 × 10^8^–5 × 10^10^ pp/mL. For an assay with dilutions 1 × 10^8^–7 × 10^9^ or less (the Target Concentration method). Limited peer-reviewed literature available	[17,18]
**Nanoparticle tracking analysis (NTA)** NanoSight NS300, Nanosight LM10 (Malvern Panalytical Ltd.)	The sample (≥200 µL) is injected into a sample chamber and illuminated by a laser beam. NPs scatter the light, which is detected using a dark field microscope. Brownian motion tracking for D_h_.	30–1000 nm	Reasonably fast (≥5 min) High-resolution particle size distribution. Can be used label-free, but compatible with fluorescence labeling → Measurements from complex sample matrices.	Limited concentration range (10^7^–10^9^) and accuracy Large sample volume Screen effect	[8,9,10,39,40]
**Tunable Resistive Pulse Sensing** **(TRPS)** qNano qViro-X Exoid (IZON Science Ltd.)	The sample (40 µL) is pipetted into a flow cell. NPs suspended in electrolytes pass through a nanopore. A change in impedance is measured for each NP. Magnitude of the signal is used to count the the particle volume. Signal frequency is used to calculate the concentration.	40 nm–20 μm	Accurate High concentration range 10^5^–10^11^ particles/mL (size dependent) and very high resolving capacity. Affordable device Measures the actual particle diameter	Approx. 10 min per sample Membranes blocked by impurities	[11,40,41]
**Flow virometry** NanoAnalyzer (NanoFCM Inc) Virus Counter 3100 (Sartorius Stedim Biotech GmbH) +Standard cytometers optimized for virus detection	Flow-cytometer optimized for NPs: Sample is streamed through a sheat-fluid containing capillary. Single-particle flow is passed through laser light beams and the scattered light or fluorescent label is observed. Labels are typically used.	Generally ≥100–200 nm when using the scatter alone, ≥20 nm when using labels NanoAnalyzer: 40–1000 nm	NanoAnalyzer: Very fast, 1 min per measure. Very high resolving capacity. Low sample volume 10–100 µL Virus Counter 3100: Easy to operate, simple no-wash staining. Virus-specific kits, available. Universal Combo Dye for enveloped viruses. Linear dynamic range of 5 × 10^5^–1 × 10^9^ vp/mL. Simultaneous analysis of light scattering and fluorescent labeling	Problems associated with fluidics, clogging and bubbles. Daily calibration needed At least medium level technical expertise needed. Generally more expensive options. Virus Counter 3100: 30 min staining + <5 min detection. Higher price-per-sample. Kits available only for a limited number of viruses. No universal stain for non-enveloped viruses. Minimum sample volume 100 µL.	[14,15,16]

## Data Availability

Not applicable.

## Appendix A

This appendix contains some of the results, according to which the preliminary LV assay was set up. The final physical particle measurement result is an average of the individual blocs analyzed. In order to set a minimum measurement length, the particle count of each bloc was compared to the final measurement result and a relative error was calculated. As a result, the minimum number of blocs to be measured was set to 5 (Figure A1a). To estimate the limit of detection (LOD), a series of blank (PBS) measurements were performed by two operators on separate days (Figure A1b). The average results from between the two operators were not different (unpaired *t*-test *p* = 0.330) and therefore readings can be combined. Based on the large measurement-to-measurement difference in the blank value, it was decided that the intermediate blank result should be followed over the following months (Figure A1c) by recording the PBS blank reading or daily average, if multiple blanks were found per day. No statistical difference (unpaired *t*-test, *p* = 0.2659) was seen between the means of daily series and the intermediate blank (7.74 × 10^7^ and 7.16 × 10^7^ pp/mL, respectively). Based on the blank results, a preliminary LOD was set to an average value of +3SD (7.16 × 10^7^ + 3 × 1.43 × 10^7^) = 1.14 × 10^8^ pp/mL. The relative error of the measurement was seen to be affected by the number of tracked particles, but with more than 40 particles tracked, the relative error drops below 10% (Figure A1d).

When measuring LV upstream samples, it is important to know the level of particles present in growth media (cell-released EVs, cell debris, etc). Cells media from two HEK293-derived cell lines were measured 2–3 days after seeding as 1:4 dilutions in PBS (our standard Videodrop starting dilution with lentivirus) (Figure A1e). The results were highly variable with zero particles tracked in some samples. According to the limited data up to 2.7 × 10^8^ pp/mL, background noise can be detected from a cell media sample.
